# TFIIS.h, a new target of p53, regulates transcription efficiency of pro-apoptotic *bax* gene

**DOI:** 10.1038/srep23542

**Published:** 2016-03-23

**Authors:** Jun-Ming Liao, Bo Cao, Jun Deng, Xiang Zhou, Michael Strong, Shelya Zeng, Jianping Xiong, Erik Flemington, Hua Lu

**Affiliations:** 1 Department of Biochemistry & Molecular Biology and Cancer Center, Tulane University School of Medicine, 1430, Louisiana, LA 70112, USA; 2 Department of Oncology, The First Affiliated Hospital of Nanchang University, Nanchang 330006, PR China; 3 Department of Pathology and Cancer Center, Tulane University School of Medicine, 1430, Louisiana, LA 70112, USA

## Abstract

Tumor suppressor p53 transcriptionally regulates hundreds of genes involved in various cellular functions. However, the detailed mechanisms underlying the selection of p53 targets in response to different stresses are still elusive. Here, we identify TFIIS.h, a transcription elongation factor, as a new transcriptional target of p53, and also show that it can enhance the efficiency of transcription elongation of apoptosis-associated *bax* gene, but not cell cycle-associated *p21 (CDKN1A)* gene. TFIIS.h is revealed as a p53 target through microarray analysis of RNAs extracted from cells treated with or without inauhzin (INZ), a p53 activator, and further confirmed by RT-q-PCR, western blot, luciferase reporter, and ChIP assays. Interestingly, knocking down TFIIS.h impairs, but overexpressing TFIIS.h promotes, induction of bax, but not other p53 targets including p21, by p53 activation. In addition, overexpression of TFIIS.h induces cell death in a bax- dependent fashion. These findings reveal a mechanism by which p53 utilizes TFIIS.h to selectively promote the transcriptional elongation of the bax gene, upsurging cell death in response to severe DNA damage.

In response to various stresses, tumor suppressor p53 transcriptionally regulates the expression of hundreds of genes associated with several essential biochemical pathways, including cell cycle arrest, apoptosis, senescence, DNA repair, autophagy, and ferroptosis^1^,^2^,^3^,^4^,^5^,^6^,^7^,^8^. Interestingly, these p53 responsive cellular effects are not induced simultaneously in response to different stresses, but rather are determined by the severity and duration of the cellular insults^9^,^10^,^11^. For example, cell cycle arrest is often required for DNA repair, while apoptosis is usually the last choice of cells to avoid transformation^9^,^12^,^13^,^14^. It is therefore logical that DNA repair- and cell cycle-associated genes should be activated at the early stage, while the apoptosis-associated target genes should be induced at the later stage upon p53 activation. Thus, the kinetics of expression of p53 targets are tightly regulated and fine-tuned to maintain homoeostasis and prevent tumorigenesis. Although posttranslational modifications of p53 and the promoter strength of target genes are thought to be associated with the selection of p53 targets at the transcription initiation and post-translation level^10^,^13^,^15^,^16^,^17^,^18^,^19^,^20^,^21^,^22^, little is known about whether selection of p53 target genes is regulated at the transcription elongation level.

TFIIS is a transcription elongation factor that is required for RNA Pol II to pass through attenuated sites, thus promoting transcription elongation^23^,^24^. TFIIS has three family members, TFIIS.o, TFIIS.l, and TFIIS.h, which are encoded in humans by the *TCEA1, TCEA2*, and *TCEA3* genes, respectively^25^. TFIIS.o is widely expressed in human tissues and is considered a “general” form of TFIIS, whereas TFIIS.l is a testis-specific isoform^23^. TFIIS.h was identified later on, and little is known about its specificity and functionality^25^. Knockout of TFIIS.o in mice causes embryotic lethality^26^, suggesting that efficient transcription elongation is vital for cellular functions. TFIIS.o also plays an oncogenic role in tumorigenesis, as knockdown of TFIIS.o suppresses proliferation and induces apoptosis in pancreatic, lung, and breast cancer cells^27^. By contrast, TFIIS.h appears to act as a tumor suppressor in cancer cells, as overexpression of TFIIS.h inhibits, while knockdown of TFIIS.h promotes, growth of ovarian cancer cells^28^. The opposite roles of TFIIS.o and TFIIS.h in cancers implicate that TFIIS.h might have different targets and act to selectively induce certain pools of genes in response to variable circumstances.

Accumulating evidence has shown that transcription elongation is not always efficient, and the RNA polymerase II (Pol II) complex could be stalled at various arrest sites with specific DNA sequences^29^,^30^,^31^,^32^. Also, it has been reported that depletion of TFIIS.o causes transcription elongation arrest at sites with specific DNA sequences. Therefore, it is possible that genes with elongation arrest sites might need another layer of regulation to facilitate their expression. These studies suggest that selection of p53 targets might also be regulated at the transcription elongation level. In this study, by treating cells with Inauhzin (INZ), a small molecule discovered in our lab as a p53 activator through ribosomal stress and/or p53 acetylation^33^,^34^, we found that TFIIS.h is a p53 target and plays a p53-dependent role in selective promotion of the transcription elongation of *bax* gene, an apoptosis-associated target of p53 35, but not of *p21*, a cell cycle-associated target^36^, providing a new insight into a novel layer of regulation of p53 targets’ expression at the transcriptional elongation level.

## Results

### TFIIS.h expression is induced by p53 activation

**Figure 1:**
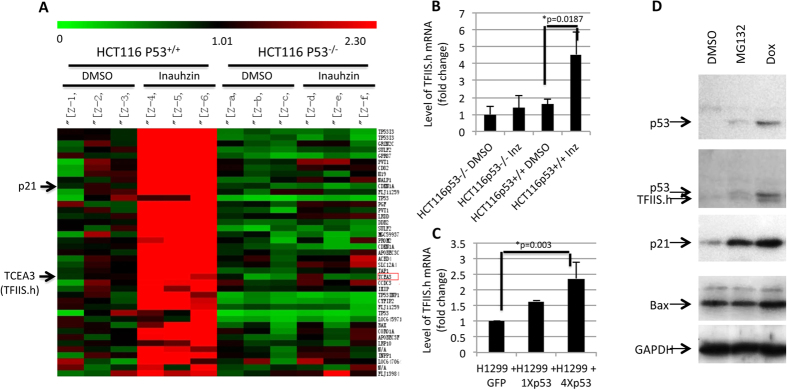
Identification of TCEA3 (TFIIS.h) as a potential p53 target gene. (**A**) *TCEA3* that encodes TFIIS.h was identified as a potential p53 target gene by gene expression profiling of HCT116 p53^+/+^ and HCT116 p53^−/−^ cells treated with a p53 activator INZ (4 μM) or DMSO (Samples were triplicated). Total RNAs were isolated from HCT116 p53^+/+^ and HCT116 p53^−/−^ cells treated as indicated and sent out for microarray analysis. (**B**) INZ induces TFIIS.h expression only in HCT116 p53^+/+^, but not in HCT116 p53^−/−^ cells. Cells were treated with 4 μM INZ or DMSO as indicated and harvested for RT-q-PCR assay to confirm the results from microarray analysis. Data are presented as means ± S.D., n = 3. (**C**) Ectopic p53 induces TFIIS.h expression dose-dependently. H1299 cells transfected with indicated plasmids were harvested and subjected to RT-q-PCT assay. Data are presented as means ± S.D., n = 4. (**D**) TFIIS.h protein expression is induced by p53 activation in response to MG132 and doxorubicin (Dox). HCT116 cells were treated with indicated drugs and harvested for WB analysis to determine the endogenous TFIIS.h protein level.

To identify novel p53 targets, we treated both HCT116 p53^+/+^ and HCT116 p53^−/−^ (without active p53) cells with or without INZ, a p53 activator identified by our lab^37^,^38^,^39^,^40^. Microarray analysis of total RNAs extracted from these samples (triplicated) showed that TFIIS.h mRNA was induced by INZ only in HCT116 p53^+/+^ cells, but not in HCT116 p53^−/−^ cells (Fig. 1A). This result was validated by RT-q-PCR analysis of RNAs isolated from the same set of cancer cells (Fig. 1B), suggesting that TFIIS.h might be a transcriptional target of p53. Next, we checked whether overexpressing p53 could induce the expression of TFIIS.h. As expected, ectopic p53 induced TFIIS.h mRNA in a dose-dependent fashion (Fig. 1C), indicating that the effect of INZ on TFIIS.h expression is due to the activation of p53 by INZ. In addition, the induction of TFIIS.h by p53 activation was further confirmed at the protein level by Western blot (WB) analysis. As shown in Fig. 1D, both bax and TFIIS.h were induced in HCT116 cells treated with doxorubicin, a p53-activating agent. However, stabilizing p53 by MG132 did not promote TFIIS.h expression that correlates well with bax (Fig. 1D), suggesting that TFIIS.h and bax might be regulated by the same signaling pathway. Together, these findings indicate that TFIIS.h is a potential p53 transcriptional target.

### p53 directly binds to TFIIS.h promoter

**Figure 2:**
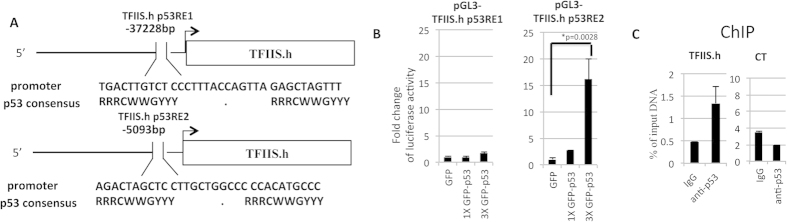
p53 binds to TFIIS.h promoter. (**A**) Schematic representation of the p53 REs upstream of the TFIIS.h gene. (**B**) p53 increases luciferase activity driven by p53RE2 found at the promoter of the TFIIS.h gene. H1299 cells were cotransfected with the indicated plasmids and β-Gal plasmid. Sixteen hours after transfection, luciferase activities were measured. Luciferase activity was normalized to β-Gal expression. Data are presented as means ± S.D., n = 3. (**C**) Endogenous p53 binds to the promoter of the TFIIS.h gene. ChIP assays were performed after HCT116 cells were treated with INZ using the indicated antibodies followed by q-PCR for TFIIS.h gene promoter sequences or negative control sequences. Data are presented as means ± S.D., n = 3.

To determine whether TFIIS.h is a direct transcriptional target of p53, we screened the upstream sequences of the TFIIS.h-coding gene and identified two putative p53 response elements (p53REs) in the promoter region using p53MH software^41^ (Fig. 2A). We then inserted these two p53REs into a pGL3 enhancer-luciferase reporter plasmid^42^ and carried out luciferase assays to test if p53 could directly bind to these two p53REs. As shown in Fig. 2B, p53 promoted luciferase activity driven by p53RE2, but not p53RE1, in a dose-dependent fashion, indicating that p53 directly binds to the p53RE2 DNA sequence located at the promoter region of the TFIIS.h gene. This result was further confirmed by a chromatin immunoprecipitation analysis (ChIP) (Fig. 2C), indicating that endogenous p53 can directly bind to the p53RE2 DNA sequence of the TFIIH.h promoter region upon activation. Taken together with the results of Fig. 1, these results demonstrate that TFIIS.h is a direct transcriptional target of p53.

### Knocking down TFIIS.h impairs the induction of bax, but not p21, by p53 activation

**Figure 3:**
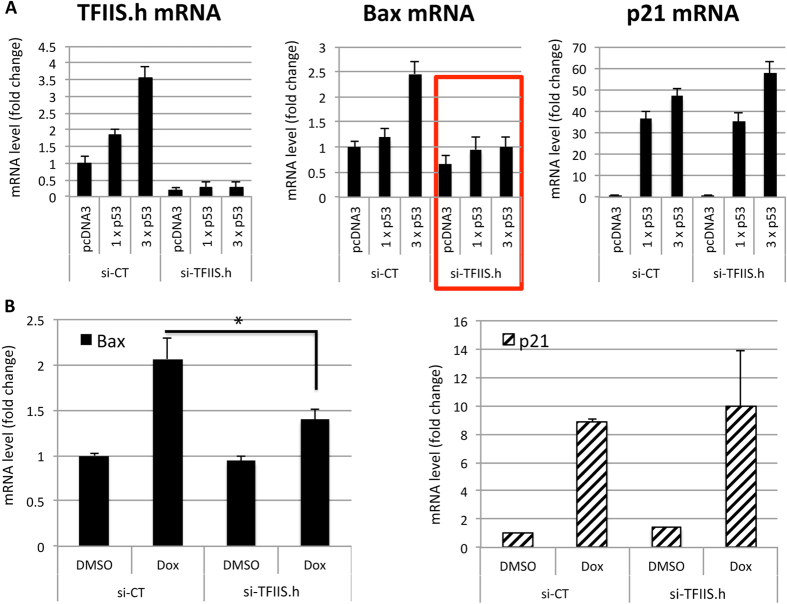
TFIIS.h knockdown affects bax, but not p21, expression. (**A**) TFIIS.h knockdown abrogates the induction of bax, but not p21, by p53. H1299 cells were transfected with indicated plasmids and harvested for RT-q-PCR assay to determine the levels of indicated mRNAs. Significance was determined using an unpaired Student’s t test. *p < 0.05. Data are presented as means ± S.D., n = 3. (**B**) TFIIS.h knockdown impairs the induction of bax, but not p21, by doxorubicin (Dox). HCT116 cells transfected with indicated plasmids were treated with 0.3 μM Dox or DMSO and harvested for RT-q-PCR to determine the mRNA levels as indicated. Significance was determined using an unpaired Student’s t test. *p < 0.05. Data are presented as means ± S.D., n = 3.

Since TFIIS.h has been reported to be required for Pol II to pass through transcription arresting sites^23^,^24^, we speculated that TFIIS.h might be involved in the transcription of some p53 target genes and thus function to selectively induce their expression associated with different cellular pathways. To test this idea, we knocked down TFIIS.h and checked the expression of several p53 target genes associated with various cellular functions in H1299 cells that were transfected with different amounts of the GFP-p53 plasmid. Surprisingly, while the expression of most tested p53 target genes, including cell cycle-associated p21^36^, autophagy-associated DRAM1^43^, and survival-associated TIGAR^44^, was not affected by depleting TFIIS.h, knocking down TFIIS.h dramatically impaired the induction of apoptosis-associated bax^35^ by p53 (Figs S1 and 3A). To further confirm that TFIIS.h is required for the induction of bax by endogenous p53 in response to DNA damage stress, we treated HCT116 cells with doxorubicin, a chemotherapy drug that causes DNA damage, and then examined the effect of TFIIS.h knockdown on bax expression. As expected, induction of bax by endogenous p53 was impaired significantly by ablating TFIIS.h, while p21 expression was not affected (Fig. 3B). These results suggest that TFIIS.h might be involved in regulation of transcription efficiency of bax.

**Figure 4:**
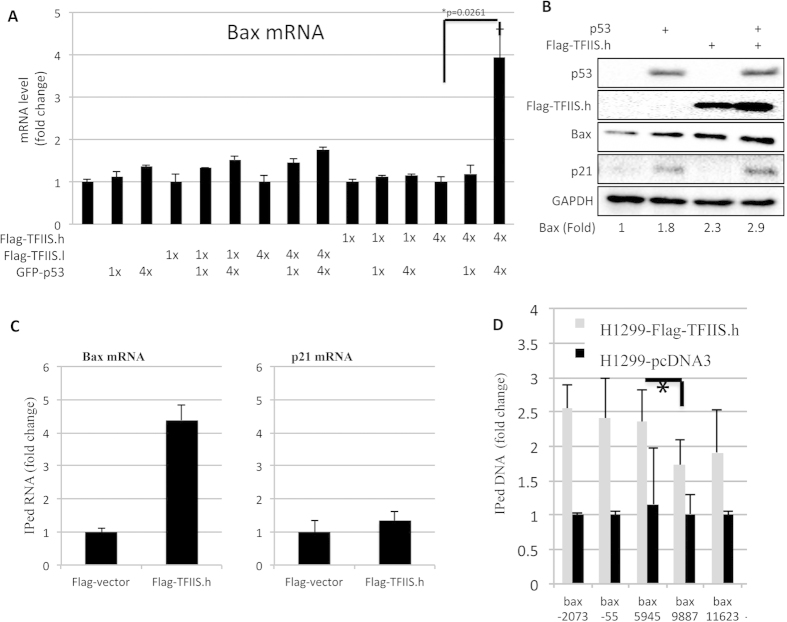
Overexpression of TFIIS.h promotes bax expression. (**A**) A high level of TFIIS.h is required for the efficient induction of bax by p53. H1299 Cells transfected with indicated plasmids were harvested for RT-q-PCR assay to check bax mRNA expression. Data are presented as means ± S.D., n = 3. (**B**) Overexpression of high level of TFIIS.h facilitates the induction of bax protein level by p53. H1299 cells transfected with indicated plasmids were harvested for WB analysis. Densitometry analysis was performed to calculate the ratio of Bas to GAPDH. Numbers depict fold change of Bax expression compared to vector control. (**C**) TFIIS.h binds to Bax nascent mRNA, but not p21 mRNA. H1299 cells transfected with indicated plasmids were harvested for RNA Immunoprecipitation Assay with flag antibody followed by RT-q-PCR to detect the indicated mRNAs. Data are presented as means ± S.D., n = 3. (**D**) TFIIS.h binds to Bax DNA. H1299 cells transfected with indicated plasmids were harvested for ChIP assay with flag antibody. DNAs pulled down by indicated antibody were determined by q-RT-PCR. Significance was determined using an unpaired Student’s t test. The difference of the levels between upstream (n = 6) and downstream (n = 4) was statistically significant. *p < 0.05. Data are presented as means ± S.D., n = 6.

Next, we tested whether p53-mediated transcription of bax is specifically regulated by flag-TFIIS.h, but not flag-TFIIS.l, by carrying out RT-q-PCR and WB after introduction of their expression plasmids with or without p53 into H1299 cells. As shown in Fig. 4A, while ectopic p53 induced the expression of bax, this induction was quite moderate, which was consistent with literature^35^. Remarkably, when titrating the TFIIS.h expression plasmid with different amounts of the p53 expression vector, this elongation factor drastically boosted the expression of bax in a dose-dependent fashion (Fig. 4A). By contrast, TFIIS.l was unable to do so (Fig. 4A). Consistently, the induction of bax protein levels by ectopic p53 was more dramatic in cells with high level of ectopic TFIIS.h than that in cells without overexpression of TFIIS.h (Fig. 4B). Again, the p53-induced expression of p21 was not elevated by ectopic TFIIS.h (Fig. 4B). These results demonstrate that TFIIS.h can promote transcription efficiency of bax, but not p21, in response to p53 and implicate that this regulation might be via transcriptional elongation.

### TFIIS.h forms a complex with bax mRNA and DNA in cells

It has been shown that TFIIS.h is recruited to transcription arresting sites and forms a complex with RNA pol II to help stalled RNA pol II pass through arrested sites^23^,^24^. Therefore, we assumed that TFIIS.h should be in the same complex of RNA pol II, bax mRNA, and DNA, if bax is a target gene of TFIIS.h. To test this idea, we transfected H1299 cells with or without flag-TFIIS.h and carried out RNA-protein complex immunoprecipitation using the anti-flag antibody. As expected, TFIIS.h only pulled down bax, but not p21, mRNA (Fig. 4C), confirming that TFIIS.h specifically regulates transcription elongation of the former, but not the latter.

Next, we speculated that TFIIS.h should be enriched at the upstream of arresting site(s) located on the *bax* gene. To test this idea, we transfected H1299 cells with flag-TFIIS.h or pcDNA3 as a control and performed ChIP assays with various primers designed for different sites of bax DNA, including −2073, −55, +5945, +9887, and +11623 from its transcription initiation site, which were used for identifying the binding sites of TFIIS.h on the bax gene. As shown in Fig. 4D, flag-TFIIS.h pulled down endogenous bax DNA, suggesting that TFIIS.h is indeed recruited to the bax gene during transcription. Interestingly, more DNAs localized in the upstream of +5945 site of the bax gene were pulled down by flag-TFIIS.h than that in the downstream sites, suggesting that the transcription arresting site of the bax gene is localized between +5945 and +9887 from initiation site. These data further confirm that TFIIS.h is recruited to the bax gene and function to promote the transcription efficiency of this gene. Notably, when we checked human cancer samples available from cBioportal, we found that TFIIS.h and bax display significant tendency towards co-occurrence in expression alteration (elevation or reduction) (Fig. S2).

### TFIIS.h promotes cell death and is downregulated in human gastric cancer tissues

**Figure 5:**
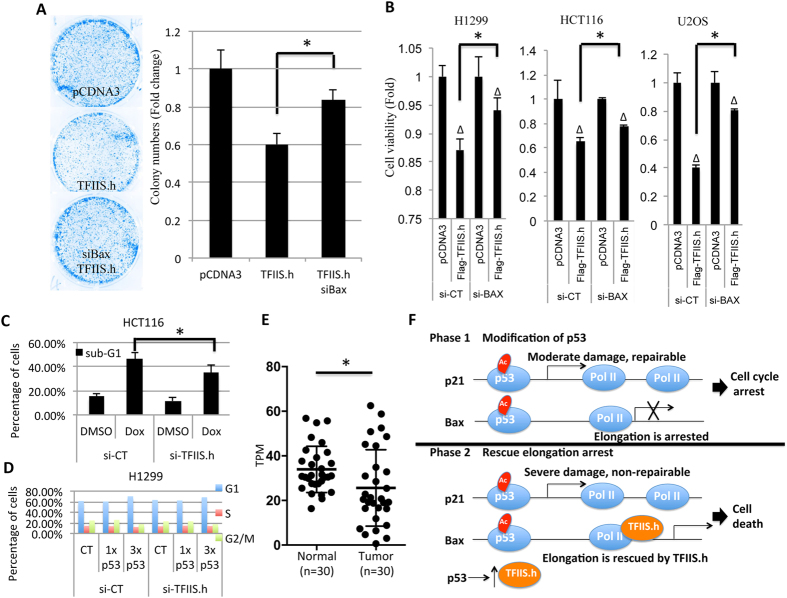
TFIIS.h promotes cell death without affecting cell cycle arrest. (**A**,**B**) Overexpression of TFIIS.h inhibits cancer cell growth in a bax-dependent manner. H1299, U2OS, or HCT116 cells transfected with indicated plasmids and/or siRNAs were subjected to colony formation assay (**A**) and cell viability assay (**B**). Data are presented as means ± S.D., n = 3. *p < 0.05. Δp < 0.05 as compared to pcDNA control. (**C**,**D**) TFIIS.h knockdown suppresses cell death, but not cell cycle arrest, induced by p53 activation. TFIIS.h knockdown impaired Dox-induced cell death. (**C**) HCT116 cells with indicated treatments were harvested (72 hours) for FACS analysis to determine sub-G1 cells. P values were determined using an unpaired Student’s t test. *p < 0.05. Data are presented as means ± S.D., n = 4. (**D**) TFIIS.h knockdown did not affect p53-induced G1 arrest. H1299 cells transfected with indicated plasmids and harvested (24 hours) for FACS analysis. (**E**) TFIIS.h is downregulated in human gastric cancers. Next generation sequencing datasets from The Cancer Genome Atlas (TCGA) initiative were downloaded from the Cancer Genomics Hub (CGHub; https://cghub.ucsc.edu) and RNA-seq datasets from 30 paired gastric adenocarcinomas and normal tissue are included for analysis^48^. *p < 0.05. Data are presented as means ± S.D. TPM: transcripts per million. (**F**) Schematic of possible selection of p53 targets in response to various stresses. Phase 1: in response to moderate damages, when cell cycle arrest is required for the recovery of cells, p53 is activated by posttranslational modifications, and thus induces the transcriptions of some cell cycle-associated genes, such as p21. However, the transcription of apoptosis-associated genes, such as bax, is arrested at specific sites to avoid unnecessary apoptosis in cells. Phase 2: in response to continuous and severe stresses, when apoptosis is required to avoid tumorigenesis, TFIIS.h is accumulated by continuously activating of p53 and acts to rescue the transcription elongation arrest of apoptosis-associated genes, such as bax.

As bax had been shown to accelerate programmed cell death^45^, we then sought to determine whether TFIIS.h could facilitate cancer cell growth inhibition or cell death. H1299 cells transfected with Flag-TFIIS.h or pCDNA3 were harvested and subjected to colony formation and cell viability assays. As shown in Fig. 5A, overexpression of TFIIS.h dramatically suppressed colony formation of H1299 cells, suggesting that TFIIS.h inhibits cell growth, which is in line with literature showing that bax induces apoptosis^45^. We then tested whether the inhibitory effect of TFIIS.h on colony formation is bax-dependent or not. Interestingly, depletion of endogenous bax by siRNAs rescued the suppression of colony formation by ectopic TFIIS.h (Fig. 5A). This result indicates that TFIIS.h functions to inhibit cancer cell growth by regulating bax expression. To further confirm this result, we performed cell viability assays using three different cell lines, H1299, HCT116, and U2OS cells. As expected, overexpression of TFIIS.h inhibited cell viability of all the tested cells (Fig. 5B). Consistently with the results from colony formation assays, the inhibitory effect of TFIIS.h on cell viability was impaired by knockding down bax, as shown in Fig. 5B, indicating that TFIIS.h promotes cell death at lease partially in a bax-dependent fashion. We noticed that knocking down bax did not completely abrogate the inhibitory effects of TFIIS.h on cell viability, which is probably due to 1) bax siRNA did not remove all the bax expression in our experiments, and 2) TFIIS.h might also regulate cell viability via bax-independent pathways. Next, we examined whether depletion of endogenous TFIIS.h could suppress cell death induced by p53 activation. To test this, we knocked down TFIIS.h in HCT116 cells treated with or without doxorubicin and carried out cell cycle analysis. Interestingly, in line with the above findings, ablation of TFIIS.h significantly impaired cell death induced by doxorubicin (Fig. 5C), suggesting that TFIIS.h is required for apoptosis induced by p53 activation. We then examined whether TFIIS.h knockdown could affect p53-induced cell cycle arrest by FACS analysis. However, we did not see significant G1 arrest in HCT116 cells treated with Dox. Therefore, we examined the effect of siTFIIS.h on G1 arrest in H1299 cells with ectopic p53. As expected, depletion of TFIIS.h had no effect on G1-arrest induced by ectopic p53 (Fig. 5D), which is in line with our finding that TFIIS.h does not regulate the expression of p21, a cell-cycle associated target of p53. These results suggest that TFIIS.h functions as a tumor suppressor during cancer development, and implies that TFIIS.h might be downregulated in human cancers. We then analyzed TFIIS.h mRNA levels in 30 paired human gastric tumor and normal tissues^46^,^47^,^48^, and found that TFIIS.h is significantly downregulated in cancer tissues compared with normal tissues, further confirming that TFIIS.h might act as a tumor suppressor in human cancers (Fig. 5E). In summary, these results demonstrate that TFIIS.h is important for bax-mediated apoptosis in response to p53 activation and may play a role as a tumor suppressor by regulating p53-dependent bax expression.

## Discussion

It has been known that p53 activates the expression of numerous transcriptional target genes that encode proteins critical for regulation of various cellular pathways or processes in response to different stresses^1^,^2^,^3^,^4^,^5^,^6^,^7^,^8^. Often these target genes are expressed to different degrees and at different time points upon p53 activation. Although comprehensive studies have proposed that posttranslational modifications of p53 and promoter strength of its target genes might be involved in the selection of p53 target genes and the regulation of their expression^10^,^13^,^15^,^16^,^17^,^18^,^19^,^20^,^21^,^22^, little is known about whether selection of p53 target genes is regulated at a transcriptional elongation level. In this study, we unraveled TFIIS.h, a transcription elongation factor that is required for pol II to pass through arresting sites^29^, as a novel transcription target of p53 (Figs 1 and 2). Surprisingly, we found that TFIIS.h binds to bax DNA and is required for bax, but not p21, induction by p53 (Figs 3, 4, 5 and S1). Also, we found that TFIIS.h promotes cancer cell death in a bax-dependent fashion (Fig. 5A–D). Furthermore, TFIIS.h was downregulated in human gastric cancers, suggesting that it is a tumor suppressor (Fig. 5E). Based on these findings, we propose that TFIIS.h is involved in selection of some p53 target genes, such as bax as shown here, at the transcription elongation level.

Although p53 target genes have similar RE sequences, the kinetics of their transcription vary dramatically^10^,^11^,^16^,^19^,^49^,^50^,^51^,^52^. Often, p53 rapidly induces genes whose proteins are important for controlling the cell cycle, but its action on the expression of apoptosis-related genes is delayed^10^. Although the biological reason for these differential actions of p53 on gene expression are generally understood, i.e., p53 might need to first rescue less genomically damaged cells through a growth arrest mechanism prior to killing them via apoptosis^10^,^50^,^51^,^53^, the mechanisms underlying the delayed transcription of apoptotic genes by p53 remain largely unknown. As depletion of TFIIS causes transcription elongation arrest at sites with specific DNA sequences^23^,^29^,^31^,^32^,^54^, it is possible that genes with elongation arrest sites might need another layer of regulation to facilitate their expression. Here, we propose that TFIIS.h might facilitate the selection of p53 targets at the transcription elongation level in response to different stresses by proposing the following possible mechanisms (Fig. 5F): Phase 1: upon moderate DNA damage or ribosomal stress^5^,^6^ when cell cycle arrest is required for the recovery of cells, p53 is induced by blocking the MDM2-p53 feedback loop to induce the transcription of p21, but the transcription of apoptosis-associated genes, such as bax, is stalled at specific sites to avoid unnecessary apoptosis; Phase 2: in response to continuous and severe DNA damaging stresses when apoptosis is required to discard severely damaged cells, TFIIS.h is induced and accumulated by continuously activated p53 to rescue the transcription elongation arrest of apoptotic genes, such as bax. Thus, TFIIS.h is another target gene of p53 and involved in promoting the induction of apoptosis-associated genes by p53. Supporting this idea is a recent study showing that TFIIS.h could unexpectedly induce apoptosis in cancer cells^28^. However, it is still largely unknown if TFIIS.h has a specific pool of target genes, which could be addressed in our future studies.

In this study, we show that TFIIS.h promotes transcription elongation efficiency of *bax* gene. As bax is a transcriptional target of p53, we speculate that TFIIS.h should specifically induce cell death in cells with active p53 functions. However, we did observe that ectopic TFIIS.h could suppress growth of H1299 cells, a p53 null cell line, in cell viability assays, although the suppression of cell growth in H1299 cells (11% suppression) was not as dramatic as those in HCT116 and U2OS cells (33% and 52% suppression), both of which express wild type p53. This could be due to the existence of other transcriptional factors controlling bax expression in H1299 cells, or other apoptosis-associated targets of TFIIS.h. Our future study identifying more specific targets of TFIIS.h by microarray analysis of cells with ectopic TFIIS.h or with depletion of TFIIS.h could address this interesting question and lead to more findings of the regulation of gene expression at the transcriptional elongation level.

In summary, our findings reveal a novel mechanism of how TFIIS.h could exert its tumor suppressive functions in facilitating p53 induction of bax. Our study not only unravels TFIIS.h as another novel bona fide target of p53, but also provides new insights into better understanding its precise regulation of the kinetics of transcription of p53 target genes associated with different cellular functions. In addition, this study also offers proof-of-concept evidence for selectively inducing apoptosis by TFIIS.h upon p53 activation, providing a potentially novel target for future identification and development of new therapies for chemo-resistant cancers.

## Experimental Procedures

### Cell lines, plasmids, and antibodies

Human HCT116 p53^+/+^ and HCT116 p53^−/−^ cells were generous gifts from Dr. Bert Vogelstein at the John Hopkins Medical Institutes. U2OS and H1299 cells were purchased from American Type Culture Collection (ATCC). All cells were cultured in Dulbecco’s modified Eagle’s medium (DMEM) supplemented with 10% fetal bovine serum (FBS), 50 U/ml penicillin and 0.1 mg/ml streptomycin at 37 °C in a 5% CO_2_ humidified atmosphere. p53 expression plasmids were described previously^55^,^56^,^57^,^58^. TFIIS.h p53 RE1 and p53 RE2 were amplified and inserted into the pGL3 Enhancer Reporter vector. TFIIS.h and TFIIS.l expression plasmids were amplified from HCT116 cells and inserted into pCDNA3-flag vector. Anti-Flag (Sigma), anti-p53 (DO-1, Santa Cruz Biotechnology), anti-actin (Sigma), anti-p21 (CP74, LifeSpan BioSciences, Inc.), anti-TFIIS.h (TCE51551, Santa Cruz Biotechnology), anti-bax (N-20, Santa Cruz Biotechnology) were commercially purchased.

### RNA preparation and microarray analysis

As described previously^39^, briefly, HCT116^p53+/+^ or HCT116^p53 −/−^ cells were treated with 4 μM INZ and harvested at 18 hours post treatment. Cells were then homogenized in TriZol reagents. Three replicates were included for each sample. Total RNA was extracted by following the manufacturer’s standard instructions (Invitrogen). RNA quality was confirmed by agarose electrophoresis and only those samples showing no degradation (ratios approaching 2:1 for the 28S and 18S bands) were sent to Arraystar Inc. (Rockville, MD), where the microarray analysis was performed on Roche/Nimblegen platform in a 12 × 135 k format. Our microarray data are deposited in GEO with accession number GSE77669.

### Transient transfection and WB analyses

As described previously^59^,^60^, briefly, cells were transfected with indicated plasmids as shown in each figure by using TransFectin (Bio-Rad), following the company’s instruction. Unless specifically mentioned, forty eight hrs post transfection, cells were harvested and lysed in lysis buffer. The total protein concentration for each sample was determined and equal amount of total proteins were then subjected to SDS-PAGE, followed by WB analysis.

### Reverse transcriptase-polymerase chain reaction and quantitative real-time PCR analysis

RT and Q-PCR for mRNAs were done by using the methods described previously^42^,^55^. Briefly, quantitative real-time PCR was performed on an ABI 7300 real-time PCR system (Applied Biosystems) using SYBR Green Mix (Applied Biosystems). Relative gene expression was calculated using the ΔC_T_ method, following the manufacturer’s instruction. All reactions were carried out in triplicate.

### Luciferase reporter assays

Cells were transfected with pGL3-TFIIS.h p53RE1 or GL3-TFIIS.h p53RE2 and indicated plasmids (total plasmid DNA 1 μg/well) as indicated in figures. Luciferase activity was determined and normalized by a factor of β-gal activity in the same assay as described previously^61^,^62^.

### Chromatin immunoprecipitation (ChIP)-PCR

ChIP analysis was performed as described previously^42^,^63^ using anti-p53 (D0-1) for endogenous p53 and anti-flag for overexpressed flag-TFIIS.h. Immunoprecipitated DNA fragments were analyzed by real-time PCR amplification using primers for indicated genes. The control primers targeting other region in the genome are as follows: 5′-AACGGTGGTGTGCGTTCCC-3′, 5′-TCTCGTCTCACTCAAACCGCC-3′.

### Cell viability assay

Cell viability assay was carried out as described previously. Briefly, cell viability was evaluated by cell counting kit (Dojindo Molecular Technologies Inc., Gaithersburg, Maryland). Cells treated as indicated were seeded onto 96-well micro plates at a density of 2 × 10^4^ cells per well. The cells were incubated WST-8 at a final concentration of 10% to each well and incubate for 2 h. Optical density (OD) was measured using a micro plate reader (Molecular Device, SpectraMax M5^e^) at 450 nm. Cell viability was calculated as a percentage of viable cells in drug-treated group versus untreated control by following equation.

Cell viability (%) = [OD (Drug)−OD (Blank)]/[OD (Control)−OD (Blank)] × 100

### Ribonucleoprotein immunoprecipitation (RNP IP) assay

RNP-IP assay was performed as described previously^59^ using the anti-flag antibody for ectopic proteins. Immunoprecipitated RNAs were extracted and analyzed by real-time PCR amplification using primers for indicated genes.

### Knockdown of endogenous mRNAs

siRNA for TFIIS.h was purchased from Santa Cruz Biotechnology. Transfection of siRNAs were performed the same as that of normal siRNA as described previously^64^ by using siLentFect^TM^ Lipid (Bio-Rad), following the manufacturer’s protocol.

### Human gastric tumor and normal tissue RNA-seq datasets

Next generation sequencing datasets from The Cancer Genome Atlas (TCGA) initiative were downloaded from the Cancer Genomics Hub (CGHub; https://cghub.ucsc.edu) and included RNA-seq datasets from normal gastric tissue (n = 33) and gastric adenocarcinomas (n = 285)^48^. 30 patients with paired normal and tumor tissues available were used for TFIIS.h expression analysis. Human gene expression was quantified using RSEM (RNA-seq by Expectation-Maximization)^65^ (using the hg19 reference genome, default parameters).

## Additional Information

**How to cite this article**: Liao, J.-M. *et al*. TFIIS.h, a new target of p53, regulates transcription efficiency of pro-apoptotic *bax* gene. *Sci. Rep.* **6**, 23542; doi: 10.1038/srep23542 (2016).

## Supplementary Material

Supplementary Information
