# Supplementary material for: TFIIS.h, a new target of p53, regulates transcription efficiency of pro-apoptotic bax gene
Source: Sci Rep. 2016 Mar 23;6:23542. doi: 10.1038/srep23542 (PMC4804275; doi:10.1038/srep23542)
Supplement: Supplementary Information [file srep23542-s1.pdf]

Supplementary Information

TFIIS.h, a new target of p53, regulates transcription efficiency of pro-apoptotic *bax* gene

Jun-Ming Liao[1], Bo Cao[1], Jun Deng[1,2], Xiang Zhou[1], Michael Strong[3], Shelya Zeng[1], Jianping Xiong[2], Erik Flemington[3], & Hua Lu[1]*

[1]Department of Biochemistry & Molecular Biology and Cancer Center, Tulane University School of Medicine, 1430, Louisiana, LA 70112, USA; [2]Department of Oncology, The First Affiliated Hospital of Nanchang University, Nanchang 330006, PR China; [3]Department of Pathology and Cancer Center, Tulane University School of Medicine, 1430, Louisiana, LA 70112, USA.

Running Title: TFIIS.h, a p53 target, regulates transcription of bax

*Corresponding Author:

Hua Lu, Department of Biochemistry & Molecular Biology and Cancer Center, Tulane University School of Medicine, 1430, Louisiana, LA 70112, Telephone: (504)-988-0394; Fax: (504)-988-1611; Email: hlu2@tulane.edu

Supplementary data

Figure S1. Screening of the p53 target genes that are affected by TFIIS.h knockdown. H1299 cells were transfected with indicated plasmids and harvested for RT-q-PCR assay to determine the levels of indicated mRNAs.

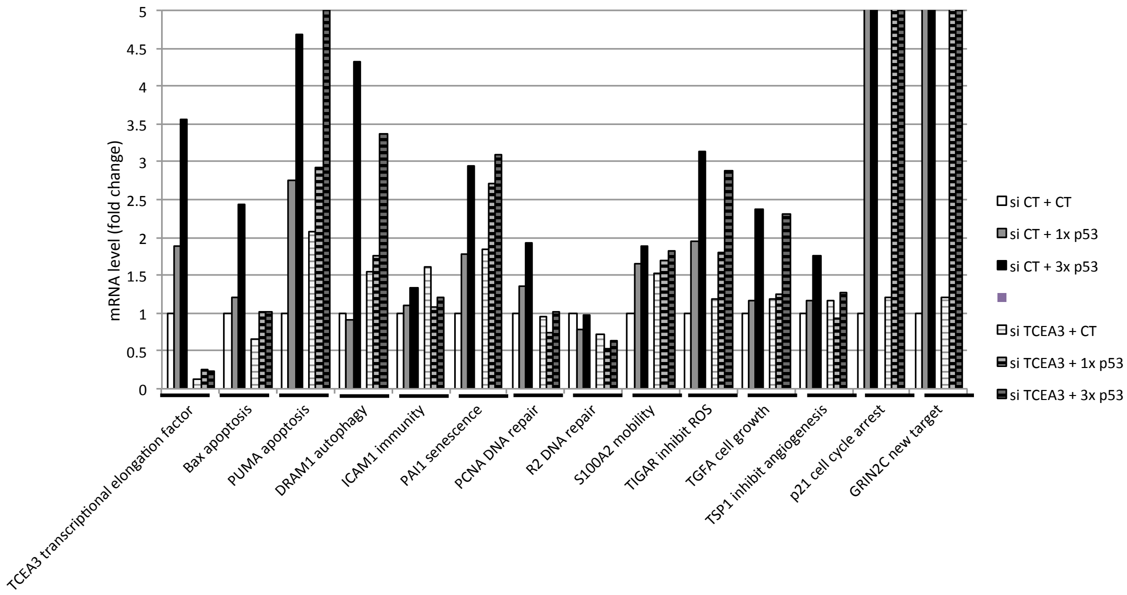

Figure S2. TFIIS.h and bax display significant tendency towards co-occurrence in expression alteration in human cancer samples available from cBioportal. Gene expression of TFIIS.h (TCEA3) and Bax in cBioportal database was analyzed, and their co-occurrence was retrieved from multiple types of human cancers as shown in the following table. P values < 0.05 suggest that the likelihood of their co-expression is statistically significant as determined by Fisher Exact Test.

Cancer type	Sample number	Log Odds Ratio	P value	Reference
**Breast Invasive Carcinoma**	1100	1.897	<0.001	**(TCGA, Provisional)**
**Cervical Squamous Cell Carcinoma and Endocervical Adenocarcinoma**	306	2.196	0.002	**(TCGA, Provisional)**
**Brain Lower Grade Glioma**	530	2.232	0.003	**(TCGA, Provisional)**
**Kidney Renal Clear Cell Carcinoma**	534	1.305	0.024	**(TCGA, Provisional)**
**Prostate Adenocarcinoma**	498	>3	<0.001	**(TCGA, Provisional)**
**Head and Neck Squamous Cell Carcinoma**	522	2.099	<0.001	**(TCGA, Provisional)**
**Head and Neck Squamous Cell Carcinoma**	279	2.006	0.007	**(TCGA, in revision)**
**Thyroid Carcinoma**	509	>3	<0.001	**(TCGA, Provisional)**
**Papillary Thyroid Carcinoma**	486	>3	<0.001	**(TCGA, Cell 2014)**
**Uterine Corpus Endometrial Carcinoma**	177	2.809	0.019	**(TCGA, Provisional)**
